# Highly drug resistant clone of *Salmonella* Kentucky ST198 in clinical infections and poultry in Zimbabwe

**DOI:** 10.1038/s44259-023-00003-6

**Published:** 2023-06-16

**Authors:** Tapfumanei Mashe, Gaetan Thilliez, Blessmore V. Chaibva, Pimlapas Leekitcharoenphon, Matt Bawn, Moses Nyanzunda, Valerie Robertson, Andrew Tarupiwa, Haider Al-Khanaq, Dave Baker, Moishe Gosa, Marleen M. Kock, Stanley Midzi, Mwamakamba Lusubilo Witson, Matheu Jorge, Jacob Dyring Jensen, Frank M. Aarestrup, François-Xavier Weill, Rene S. Hendriksen, Marthie M. Ehlers, Robert A. Kingsley

**Affiliations:** 1 University of Pretoria, Pretoria, South Africa; 2 National Microbiology Reference Laboratory, Harare, Zimbabwe; 3 World Health Organization, Harare, Zimbabwe; 4 Quadram Institute Bioscience, Norwich, UK; 5 Ministry of Health and Child Care, Harare, Zimbabwe; 6 Technical University of Denmark, Kgs. Lyngby, Denmark; 7 Earlham Insitute, Norwich, UK; 8 Irvines, Harare, Zimbabwe; 9 University of Zimbabwe, Harare, Zimbabwe; 10 National Health Laboratory Service, Pretoria, South Africa; 11 World Health Organization Regional Office for Africa, Brazzaville, Republic of Congo; 12 World Health Organization, Geneva, Switzerland; 13 Institut Pasteur, Paris, France; 14 University of East Anglia, Norwich, UK; 15 Present Address: University of Leeds, Leeds, UK

**Keywords:** Pathogens, Bacterial genes

## Abstract

A highly multidrug-resistant strain of *Salmonella enterica* serotype Kentucky (*S*. Kentucky) of sequence type (ST)198 emerged in North Africa and has since spread widely. To investigate the genetic diversity and phylogenetic relationship of *S*. Kentucky in Zimbabwe and identify potential sources of infection, the whole-genome sequence of 37 *S*. Kentucky strains isolated from human clinical infections and from poultry farms between 2017 and 2020 was determined. Of 37 *S*. Kentucky isolates, 36 were ST198 and one was ST152. All ST198 isolates had between six and fifteen antimicrobial resistance (AMR) genes, and 92% carried at least ten AMRs. All ST198 isolates harbored the *Salmonella* genomic island K-Israel variant (SGI1-KIV) integrated into the chromosome with *aac(3)-ld, aac(6)-laa, aadA7*, *bla*_TEM-1_, *sul1*, and *tetA* genes, with occasional sporadic loss of one or more genes noted from five isolates. All ST198 isolates also had mutations in the quinolone resistance-determining region of the *gyr*A and *parC* genes. The *bla*_CTX-M-14.1_ and *fos*A3 genes were present in 92% of the ST198 isolates, conferring resistance to extended-spectrum cephalosporins and fosfomycin, respectively, were present on an IncHI2 plasmid with the *aadA2b, aadA1, aph(3’)-Ib, aph(6’)-Id, cmlA1* and *sul3* AMR genes. *S*. Kentucky ST198 isolates from Zimbabwe formed a closely related phylogenetic clade that emerged from a previously reported global epidemic population. The close genetic relationship and population structure of the human clinical and poultry isolates of ST198 in Zimbabwe are consistent with poultry being an important source of highly resistant strains of *S*. Kentucky in Zimbabwe.

## Introduction

The global spread of antimicrobial-resistant bacteria including high-risk clones has been described as one of the greatest threats facing humankind in the 21st century^1^, with an estimated 1.27 million deaths per year attributable to bacterial antimicrobial resistance (AMR)^2^. The prevalence of AMR in low-income countries is generally greater than that in high-income countries^3^ and poor health care provision in these countries contributes to their vulnerability to infection. Factors leading to the spread of resistance are complex but primarily attributed to the overuse of antibiotics in clinical and agricultural practice^4^, and response, several national initiatives have been implemented to promote the responsible use of antimicrobials in animal production^5^. Antibiotics are commonly used therapeutically or as growth promotors in intensive livestock production systems resulting in the emergence of resistant bacteria that can rapidly spread between animals and farms and into the food chain^6^. Food is one of the most important transmission pathways for AMR pathogens from livestock to humans^6^, although the direct transfer to farm workers and veterinarians has also been described^7^. Treatment of human clinical infections with antibiotics may also select for AMR that can transmit to animal populations via sewage^8^. To combat the threat to human health from antimicrobial resistance, an understanding of the mechanisms of resistance and the drivers of its emergence is needed^4^.

Non-typhoidal *Salmonella* (NTS) serotypes are associated with a significant public health burden worldwide. Although commonly a self-limiting gastroenteritis with low case fatality rate and antibiotic treatment is contraindicated, infections resistant to ampicillin, chloramphenicol, streptomycin, sulfonamide, tetracycline, and quinolone antibiotics were associated with increased morbidity and mortality in Denmark^9^. A severe invasive non-typhoidal *Salmonella* (iNTS) disease may occur in immunocompromised people due to coinfections or at the extremes of age requiring treatment with antibiotics^10^. Multidrug-resistant (MDR) strains of *Salmonella enterica* serotype Typhimurium (*S*. Typhimurium) and *S*. Enteritidis are commonly associated with iNTS disease in sub-Saharan Africa^11–13^. There are no specific recommendations for the treatment of iNTS, but in sub-Saharan Africa infections are commonly treated with Fluoroquinolones or extended-spectrum cephalosporins, where available^14^. The recent emergence of strains resistant to fluoroquinolones due to mutations in the *gyrA* and *parC* genes or extended-spectrum cephalosporins through the expression of extended-spectrum beta-lactamase has reduced the treatment options for human infections^15,16^.

*S*. Kentucky infections have been commonly linked to the consumption of contaminated poultry globally^17^ and may acquire resistance particularly easily in response to selection pressure exerted by the use of antibiotics^18^. *S*. Kentucky was first isolated from a chicken in the United States of America (USA) in 1937^19^. Although most infections produce mild gastroenteritis, life-threatening disseminated infections are atypically common among elderly and immune-compromised patients compared to other serotypes^20^. Antimicrobial resistance has been particularly associated with a clone of sequence type (ST) ST198 that emerged in Egypt around the year 1989 and spread across Africa, into Europe, the Middle East and Asia^17^. Multidrug resistance in ST198 is encoded on *Salmonella* genomic island 1 (SGI1)^21^, an integrative mobilizable element that harbors a gene cluster^22^ conferring resistance to ampicillin, chloramphenicol, streptomycin, sulphonamides, and tetracyclines^23^. SGI1 with variable gene complement and arrangement such as SGI1K^17^ and SGI1-KIV^24^ has been identified in multiple *Salmonella* serotypes and strains. *S*. Kentucky ST198 has continued to evolve ever greater resistance, notably to fluoroquinolones then to extended-spectrum cephalosporins. Resistance to fluoroquinolone antibiotics due to mutations in the *gyrA* and *parC* genes was first reported in France by a traveler returning from Egypt in 2002^17,25^. Subsequently, 74% of *S*. Kentucky isolates from 12 countries between 2007 to 2012 were resistant to ciprofloxacin^26^. Extended-spectrum β-lactamase (ESBL) producing ST198 was originally imported to Europe via travelers returning from North Africa^20^ and may have been established in some regions of Europe^18^.

The molecular epidemiology and extent of ESBL-producing *S*. Kentucky has been reported in several European countries^18,27^ but remain unknown for Zimbabwe. In this, study, the population structure of isolates recovered from human clinical infections, farm workers, poultry, the poultry farm environment, and poultry feed in Zimbabwe using whole-genome sequencing (WGS) were investigated. Furthermore, we investigated the distribution and genetic flux of AMR determinants of strains identified in Zimbabwe.

## Results

### *S*. Kentucky is a common serotype isolated from poultry and human clinical infections in Zimbabwe

To identify *S. enterica* strains associated with poultry and human clinical infection in Zimbabwe, the whole-genome sequence for 245 non-typhoidal *Salmonella* strains isolated during routine clinical diagnostics surveillance or from a chicken farm surveillance study, was determined. In silico prediction of serotype using whole-genome sequence revealed a total of 44 distinct serotypes, included 42 *S*. Enteritidis (17%), 37 *S*. Kentucky (15%), 22 *S*. Heidelberg (9%), and 17 *S*. Typhimurium (6.9%), together accounting for approximately half of all isolates (Supplementary Fig. 1). *S*. Kentucky represented the most commonly isolated serotype from poultry and farm environment and the fifth most common from human clinical cases of infection. Of 37 *S*. Kentucky isolated, 11 were from human clinical infections from Harare city (7/11, 64%), and one each from Kadoma, Chitungwiza, Mutare and Chiredzi, from the years 2017 to 2019 (Supplementary Data). Seven cases (64%) were female and four (36%) male, ranging in age from nine months to 76 years, with the majority of cases (55%) in persons under 15 years of age. In all cases, isolation of the bacteria was from stool. Among the 26 isolates from chicken farms, 15 were from chickens, eight from the chicken farm environment, two from farm personnel and one from chicken feed (Supplementary Data).

### Phylogenetic relationship and molecular epidemiology of *S*. Kentucky in Zimbabwe

**Fig. 1:**
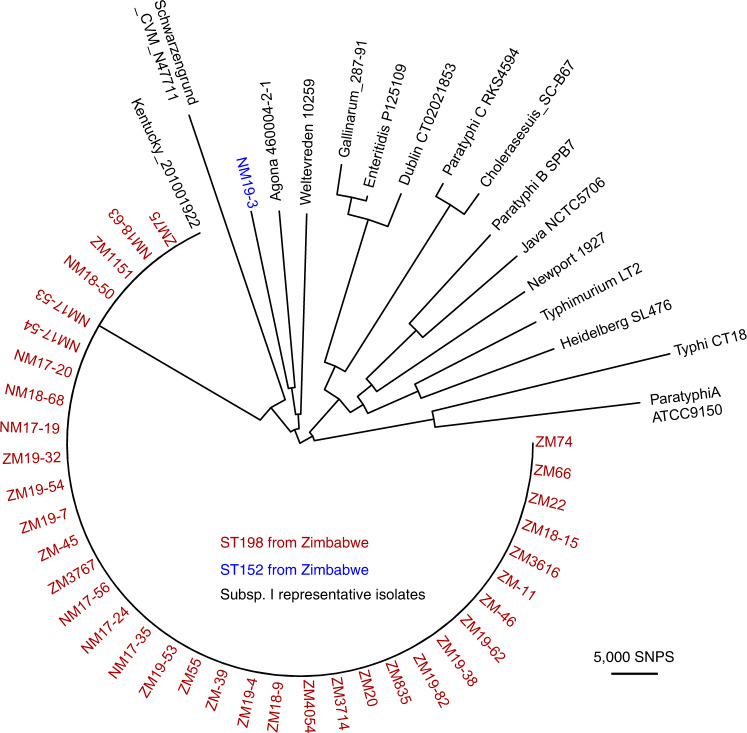
Phylogenetic relationship of *S*. Kentucky strains isolated in Zimbabwe in the context of representative strains of subspecies I. Maximum likelihood phylogenetic tree constructed using sequence variation in the core genome of 37 *S*. Kentucky strains isolated in Zimbabwe and fifteen reference strains of diverse *S. enterica* subspecies I serotypes rooted on *Salmonella bongori* ASM25299v1 as the outgroup (not shown). The position of *S*. Kentucky ST198 (red taxa labels) and ST152 (blue taxon label) and representative strains of diverse serotypes (black taxa labels) are shown.

To investigate the phylogenetic relationship of 37 strains, we first determined the sequence type. A total of 36 strains belonged to ST198 (97.3%) and a single isolate belonged to ST152 (2.7%) (Fig. 1). To investigate the phylogenetic relationship of the isolates from Zimbabwe in the context of fifteen serotypes of *Salmonella enterica* subspecies I, a maximum likelihood tree was constructed based on sequence variation in the core genome (Fig. 1). All 36 of the ST198 strains isolated in Zimbabwe clustered together in a clade along with the ST198 reference strain 201001922. In contrast, the ST152 strain belonged to a distinct lineage with a similar level of genetic divergence to other serotypes investigated, indicating that ST198 and ST152 acquired the same O-antigens by convergent evolution (Fig. 1). As ST198 is the main sequence type found in Zimbabwe and an epidemic clone of this ST was previously reported^17^, further analysis was focused on the 36 ST198 strains.

### ST198 strains isolated in Zimbabwe from human clinical infection are closely related to poultry isolates

**Fig. 2:**
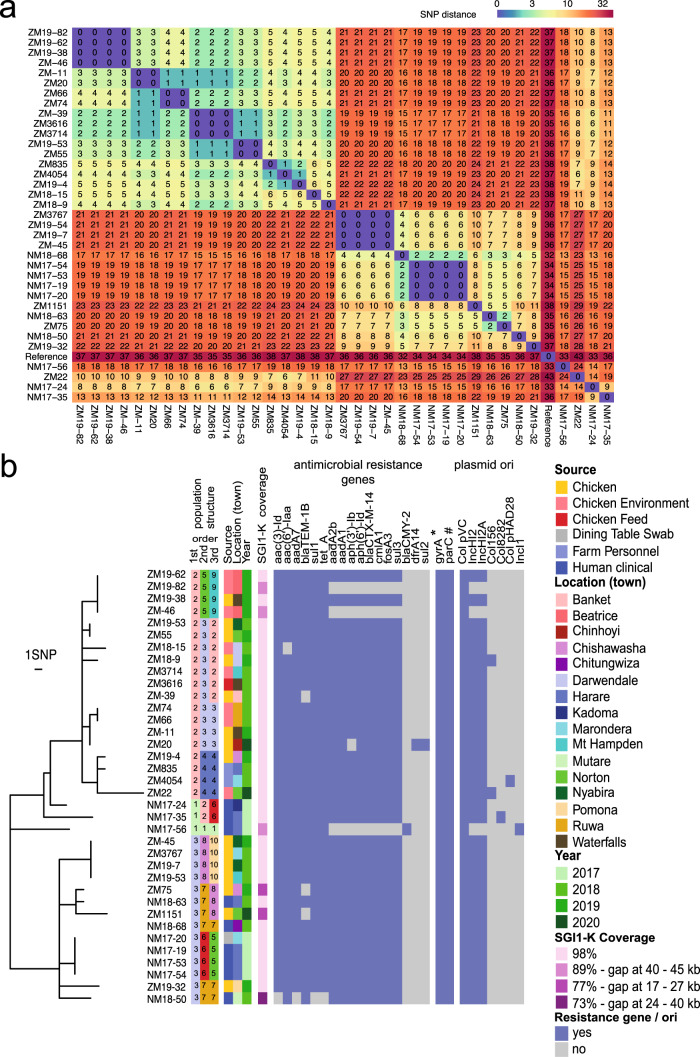
Genetic diversity and population structure of *S*. Kentucky ST198 strains isolated in Zimbabwe. **a** Pairwise single-nucleotide polymorphism (SNP) matrix of 36 *S*. Kentucky ST198 isolates from human and animal sectors isolated from 2016 to 2020 in Zimbabwe. **b** Maximum likelihood phylogenetic tree constructed based on nucleotide sequence variation in the shared genome sequence with reference to the whole-genome sequence of strain 201001922 (GenBank accession number CP028357). The tree was rooted on *S*. Typhimurium SL1344 as the outgroup (not shown). The population structure organized into eleven clades based on three orders are indicated by integers and colored blocks, and the source, location, year of isolation and the presence of antimicrobial or plasmid replicon genes are color coded as indicated in the inset key. The presence of mutations resulting in S83F and D87Y substitutions in *gyrA* (*) or S80I in *parC* (#) are indicated.

Pairwise comparison of single-nucleotide polymorphisms (SNPs) of the 36 ST198 strains from Zimbabwe indicated a mean root-to-tip distance of ~12 SNPs, consistent with a recent common ancestor (Fig. 2a). The population structure based on shared and unique SNPs indicated three first-order clades, eight second order and ten third-order clades (Fig. 2b). First-order clade 1 comprised three basal-rooted human clinical isolates, clade 2 contained isolates from chickens, the chicken farm environment and farm workers and clade 3 contained human clinical isolates in addition to farm isolates. Several poultry and human isolates differed by fewer than five SNPs, consistent with potential recent transmission events^28^. However, these were from a different geographical location within Zimbabwe or different years of isolation, consistent with recent spread within Zimbabwe. For example, clade 2.4.4 contained four isolates from chickens, a farm environment, and two farm workers. Isolate ZM19-4 from a chicken had one and two SNPs compared with strains ZM4054 and ZM835, respectively, that were isolated from farm workers. Similarly, a clinical isolate, NM18-63 in clade 3.7.8 differed from the two chicken isolates, ZM75 and ZM1151, by two and five SNPs, respectively (Fig. 2a). Closely related poultry, environmental and human isolates come from different times and geographical locations in Zimbabwe, was consistent with recent spread of the epidemic strain rather than direct transmission. In addition, strain NM17-20 in clade 3.6.5, was isolated from a dining table in Marondera and was identical to three human clinical isolates from Harare in the same year, suggesting contamination from a shared source (Fig. 2b). The population structure is also consistent with inter-farm transmission of *S*. Kentucky, as evidenced by four identical strains in clade 3.8.10 that originated from chickens on farms in Nyabira, Marondera, and Mt Hampden (Fig. 2b).

### ***S****.* Kentucky ST198 from Zimbabwe encode resistance to a broad range of antimicrobials

All ST198 strains isolated in Zimbabwe contained at least six AMR genes, and 92% contained a total of between ten and fifteen AMR genes. Most strains (86%) had an *aadA7*, *bla*_TEM-1_, *sul1*, and *tetA* gene known to be associated with SGI-1 in S. Kentucky ST198 strains^17^ and 92% also had *aadA*, *aph(*6)-ld, *bla*_CTX-M-14.1_, *cml*, *fosA3*, and *sul3* genes. Together these AMR genes were predicted to confer resistance to diverse classes of antibiotics including aminoglycosides, β-lactams, fosfomycin, phenicol, quinolones, sulphonamides, and tetracycline. In addition, fluoroquinolone resistance was due to point mutations in the chromosomal genes *gyr*A and *parC* (Fig. 2b).

A wide range of plasmid replicons were present in both clinical and poultry farm strains, of which *ColpVC* and Inc*HI2*/ Inc*HI2A* were the most abundant (36/36 or 100% and 33/36 or 92% isolates, respectively). The presence of the *aadA*, *aph(*6)-ld, *bla*_CTX-M-14.1_, *cml*, *fosA3*, and *sul3* in 92% of strains coincided with the presence of an Inc*HI2* origin of replication. The presence of these resistance genes in deeply rooted lineages was consistent with their acquisition by a common ancestor of ST198 strains from Zimbabwe and occasional sporadic loss of between one and eight genes in three strains (ZM19-82, ZM-46, and NM17-56) (Fig. 2b).

Strain NM17-56 contained a *bla*_CMY-2_ gene that coincided with the presence of an IncI plasmid origin of replication. Strain ZM1151 contained the *qnr*B gene conferring decreased susceptibility to fluoroquinolone antibiotics that was also the only strain in this collection that did not have mutations in the *gyrA* gene which is associated with resistance to these antibiotics. Finally, strain ZM20 had the *dfr*A14 and *sul*2 genes that was not accompanied by additional plasmid replicons in available databases (Fig. 2b).

### Antimicrobial resistance is associated with plasmids and an integrative mobilizable element SGI1 in the Zimbabwe *S*. Kentucky ST198

**Fig. 3:**
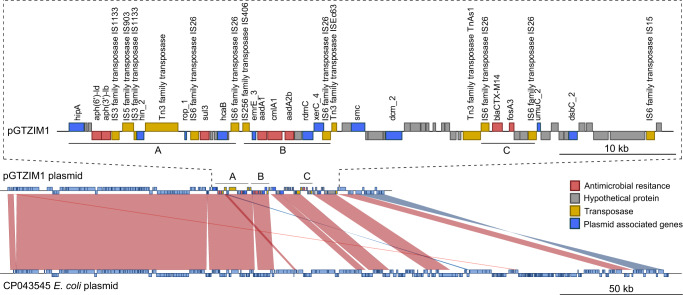
Alignment of IncHI2 plasmid pGTZIM1 carrying a *bla*_CTX-M14_ with its closest relative in the available database. Nucleotide sequence (horizontal lines) of pGTZIM1 and *E. coli* plasmid pF218CHI2 (accession CP043545) and predicted open reading frames (blue boxes) are represented. Nucleotide sequences with >90% identity over >900 bp are indicted by red shading. A region of pGTZIM1enclosed in a dashed line box is details regions A and B carrying *aph(*6), *bla*_CTXM-14.1_, *cml*, *fosA3* and *sul3* genes. Boxes are color-coded based on putative function and annotation based on alignment (inset key).

To further investigate the co-occurrence of Inc*HI2* replicon genes with *aadA*, *aph(*6), *bla*_CTX-M-14.1_, *cml*, *fosA3,* and *sul3* AMR genes and an Inc*I* plasmid carrying the *bla*_CMY_ gene, the complete and closed whole-genome sequence of strains NM17-19 and NM17-56 was determined using long-read sequencing. A contiguous assembled sequence of approximately 157 kb containing an IncHI2 replicon (PTU-HI2) and the *aad*A, *aph(*6)-ld, *bla*_CTX-M-14.1_, *cml*, *fosA3* and *sul3* resistance genes, present on a composite transposon was identified and designated pGTZIM1 (Fig. 3). Alignment of the sequence to the PLSDB plasmid database indicated that a plasmid pF218CHI2 (accession NZ_CP043545.1) from an *E. coli* strain as the closest known relative. Plasmid pF2_18C_HI2 also carried the *aad*A, *aph(*6), *cml* and *sul*3 resistance genes found in plasmid pGTZIM1, but lacked the *bla*_CTX-M-14.1_ and *fosA3* genes. The *bla*_CTX-M_ gene present in pF2_18C_HI2 differed from *bla*_CTX-M-14.1_ by a non-synonymous mutation resulting in a predicted I17F substitution in the primary amino acid sequence (Supplementary Fig. 2).

**Fig. 4:**
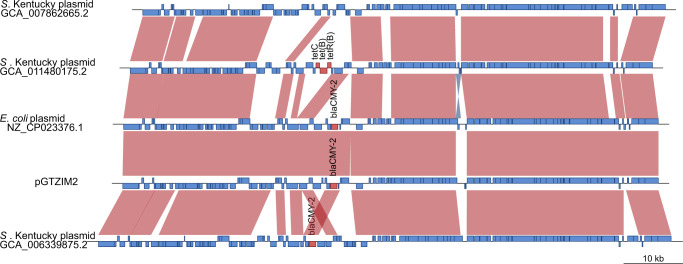
Comparison of IncI plasmid pGTZIM1 carrying a *blaCMY-2* to its closest relatives in the plasmid database. Nucleotide sequence (horizontal lines) of pGTZIM2 and *E. coli* plasmid p92 (accession NZ_023376.) and three plasmids (GCA_007862665.2, GCA_011480175.2 and GCA_006339875.2) isolated from *Salmonella* Kentucky and predicted open reading frames (blue boxes) or predicted AMR genes (red boxes) are represented. Nucleotide sequence with >90 over >900 bp are indicated by red shading.

A second contiguous assembled sequence of 92.5 kb from NM17-56 contained an IncI replicon (PTU-I1) and carried a *bla*_CMY_ gene and was designated pGTZIM2 (Fig. 4). Alignment of the sequence to the PLSDB plasmid database indicated that plasmid p92 (RefSeq NZ_023376.1) first identified in an *E. coli* strain was the closest known relative. Three other plasmids from *S*. Kentucky strains in the database were also close relatives, sharing the same backbone, but only one had a *bla*_CMY_ gene (GCA_006339875.2), a second carried the *tetC*, *tet*, and *tetR* tetracycline resistance genes (GCA_011480175.2), while the third lacked resistance genes (GCA_007862665.2). The long-read assembly of both NM17-19 and NM17-56 revealed the presence of a 3.3 kb ColpVC plasmid (PTU-E1), which also shares similarities with an *E. coli* plasmid (pCFS3313-4, RefSeq accession number NZ_CP053654.1), that we designated pGTZIM3 (Supplementary Fig. 3). Alignment of the sequence to available databases failed to identify known AMR or virulence genes in pGTZIM3.

### ST198 from Zimbabwe carry SGI1-KIV

**Fig. 5:**
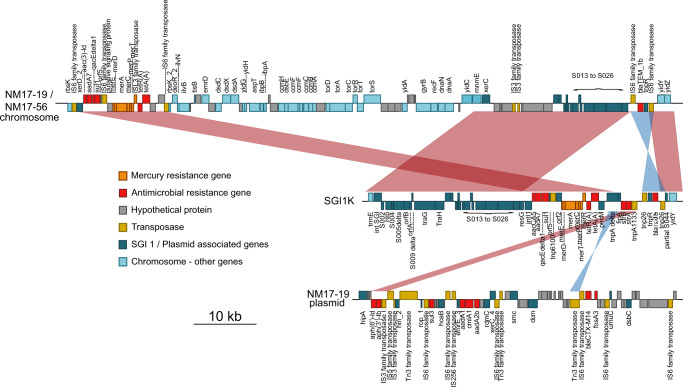
Alignment of the nucleotide sequence of SGI1-K to SGI1-KIV present in *S*. Kentucky isolates Zimbabwe. sequence (horizontal lines) of SGI1-KIV, canonical SGI1-K and pGTZIM1, with predicted open reading frames of *S*. Kentucky chromosomal genes and SGI1 genes color coded as indicated in the key (inset). Nucleotide sequence with >90% identity over >900 bp are indicated by red shading.

Mapping of short-read sequence of *S*. Kentucky ST198 strains from Zimbabwe to SGI-1K (accession AY463797.8) indicated the presence of an SGI1-K-like element. Most isolates had >98% coverage of SGI-1K and the remaining six had greater than 73% coverage with various potential deletions (Fig. 2b). Alignment of the long-read genome assemblies of NM17-19 and NM17-56 revealed a genomic structure of SGI1 different from the canonical SGI1-K and identical to a previously reported SGI1K variant, designated SGI1-KIV (SGI1-K Israeli Version, Fig. 5)^24^. Unlike SGI1-K which is present as a single contiguous insertion in the *trmE*/*ydiY* locus, SGI1-KIV was present in two sections inserted into the *rbsK* locus and *trmE*/*ydiY* loci separated by 50 kb of the core genome sequence. Furthermore, SGI1-KIV lacked the *aph*(6’) and *aph*(3’) resistance genes present on the canonical SGI1-K, although in strains NM17-19 from Zimbabwe, the *aph*(6’) and *aph*(3’) were present on the IncHI2 plasmid pGTZIM1 (Fig. 5).

### ST198 strains from Zimbabwe are part of an internationally dispersed MDR clone

**Fig. 6:**
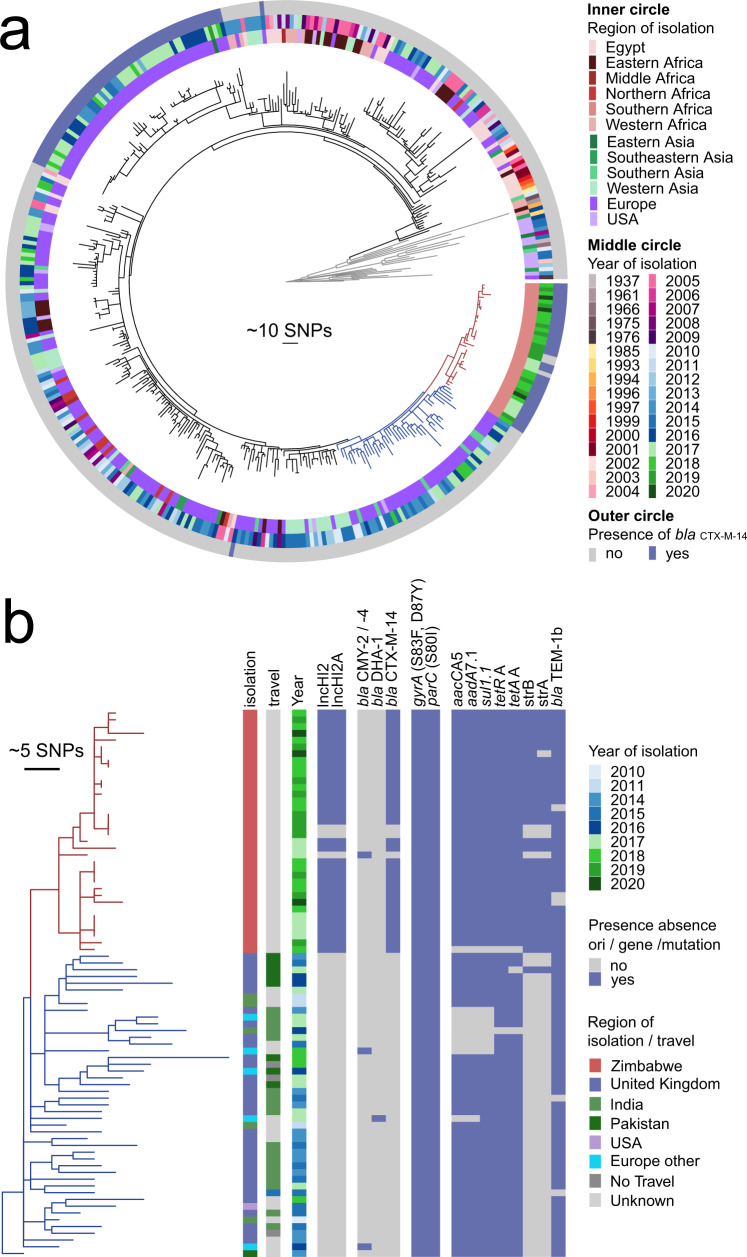
Population structure of *S*. Kentucky ST198 strains isolated from Zimbabwe in the context of 364 globally sourced *S*. Kentucky strains. **a** Maximum likelihood phylogenetic tree constructed based on recombination-purged SNPs in the shared genome with reference to *S*. Kentucky strain 201001922 (GenBank accession number CP028357). The tree was rooted on *S*. Typhimurium SL1344 strain SL1344 as an outgroup (not shown). The region (inner circle) and year (middle circle) and presence of a *bla*_CTXM-14_ gene (outer circle) are indicated by colors (inset key). Deeply rooted lineages (gray lines), lineages of strains isolated from Zimbabwe (red lines) and closely related strains isolated elsewhere (blue lines) are indicated to assist interpretation. **b** A subtree extracted from that shown above including strains isolated from Zimbabwe (red lines) and closely related strains isolated elsewhere (blue lines). Colored boxes indicate the country of isolation and country implicated through recent travel, if known (inset key), and the presence of AMR genes are indicated (inset key).

The phylogenetic relationship of 36 *S*. Kentucky ST198 strains isolated in Zimbabwe were investigated in the context of 364 ST198 strains isolated in 33 countries on five continents between 1937 and 2020. A maximum likelihood phylogenetic tree constructed based on recombination-purged sequence variation in the core genome revealed a population structure with multiple deeply rooted clades (Fig. 6a). Most of the deeply rooted branches consisted of a single isolate on long extended branches that were predominantly isolated from the US or Southern and East Asia (gray lineages in Fig. 6). A single deeply rooted lineage gave rise to a clade containing the majority of *S*. Kentucky ST198 strains. This large clade contained strains isolated from many countries worldwide, but strains present in a basal clade and therefore most closely related to the hypothetical ancestor were predominantly from Egypt. ST198 strains isolated in Zimbabwe formed a distinct subclade that was nonetheless closely related to consisting of 29 closely related ST198 strains from UK (33 strains), India (5 strains), Denmark (2 strains), Pakistan (1 strain), Netherlands (1 strain), US (1 strain) and Belgium (1 strain) (Fig. 6b). However, 25 of the strains isolated in the UK were associated with travel to India or Pakistan, while travel information for six isolates was not known. Therefore 31 of 45 (68%) of strains were isolated from or known to be associated with travel to South Asian Countries, implicating spread from these countries to Zimbabwe. A *S*. Kentucky ST198 strain isolated in Israel in which SGI1-KIV was first reported was present in a more deeply rooted clade than the Zimbabwe clade (Fig. 6a).

Determination of the presence of AMR genes in the global collection of *S*. Kentucky ST198 indicated that isolates from Zimbabwe contained more AMR genes in part due to the acquisition of pGTZIM1 (Supplementary Fig. 4, Fig. 6b, and Supplementary Data). The *aad*A7, *bla*_TEM-1_, *sul*1 and *tetA* genes, commonly associated with SGI1, were present in most ST198 strains from the global collection, consistent with its acquisition immediately prior to clonal expansion and spread as previously reported. In contrast, the *aph(*6), *bla*_CTXM-14.1_, *cml*, *fosA3*, and *sul3* genes present in the majority of ST198 strains from Zimbabwe on the IncHI2 plasmid pGTZIM1, were generally absent from strains isolated from elsewhere, consistent with recent acquisition potentially within Zimbabwe or an unsampled population giving rise to the Zimbabwe subclade. Sporadic distribution of a subset of *aph(*6), *bla*_CTXM-14.1_, *cml*, *fosA3*, or *sul3* genes were present in individual strains or clusters of strains isolated from outside of Zimbabwe, and all but three of strains lacked an IncHI2 origin of replication. Conversely, three strains isolated from outside of Zimbabwe had the IncHI2 origin of replication and at least one of the *aph(*6), *bla*_CTXM-14.1_, *cml*, or *sul3* genes while six had none of these genes.

## Discussion

Diverse NTS serotypes were isolated through routine surveillance of human clinical infections and poultry-associated sources between 2016 and 2020 in Zimbabwe. A total of 45 different serotypes were represented among 245 isolates. Overall, *S*. Kentucky was the second most frequently isolated serotype, representing 15.1% (37/245) of the total isolates. Over-representation of poultry-associated sources in this study is likely to have contributed to elevating the frequency of isolation of the *S*. Kentucky that is particularly common in this host species^29^. Nonetheless, *S*. Kentucky was the 5^th^ most frequently isolated serotype from human clinical infection in this strain collection from Zimbabwe and is therefore a serotype of significant concern to public health. With the notable exception of invasive disease where *S*. Typhimurium and *S*. Enteritidis dominate, few studies have reported the relative frequency of NTS serotypes in clinical infection or from livestock in sub-Saharan Africa and *S*. Kentucky has not been reported as common^10^. *S*. Kentucky was reported as relatively common in gastroenteritis NTS infections in North Africa and the Middle East^30^. This study is therefore the first to report WGS analysis of ESBL-producing *S*. Kentucky strains of human and poultry origin in Sub-Saharan Africa.

All but one of the NTS strains from Zimbabwe investigated in this study belonged to ST198, with a single strain belonging to ST152. The presence of these sequence types on distinct long basally rooted lineages in the population structure of *S. enterica* subspecies I indicated that the serotype is polyphyletic, with the antigens used to define serotypes emerging independently as observed for some serotypes such as *S*. Derby and *S*. Paratyphi B^31,32^. The low number of SNPs within the ST198 cluster was consistent with a recent common ancestor within the past decade based on published molecular clock rates of ~1–2 SNPs per genome per year for *Salmonella* epidemic clades^12,33,34^. This lack of genetic diversity and wide geographical distribution within Zimbabwe suggests that the clone has spread rapidly to many farms across the country. The presence of strains of *S*. Kentucky ST198 in feed that were closely related to strains isolated from poultry implicates this as a potential source of transmission. Nonetheless, the relative contribution of livestock transfer, other animal species or environmental factors and feed in the transmission of *S*. Kentucky ST198 between farms in Zimbabwe cannot be assessed with these data. The close genetic distance between isolates are also consistent transmission of *S*. Kentucky from poultry to humans, but due to a small dataset and the limitation of sampling, no case of direct transmission could be inferred with high confidence.

Strains isolated in Zimbabwe formed a distinct clade within a globally dispersed ST198 population that emerged in Egypt in 1989 and was associated with multidrug resistance conferred by the acquisition of SGI-1 and resistance to fluoroquinolones due to mutations in the *gyrA* and *parC* genes^17,35–42^. The Zimbabwe clade was distally rooted within the phylogeny of globally sourced strains of ST198, suggesting that this clone spread to Zimbabwe later than those in other countries represented in the global collection. Consistent with this idea, all the Zimbabwe strains contained resistance genes present in SGI-1 and mutation substitutions in the *gyrA* and *parC* genes known to confer resistance to ciprofloxacin^36^.

The prevalence of ESBL-producing *S*. Kentucky in Zimbabwe is concerning as extended-spectrum cephalosporins are currently the first-line antimicrobials for the empiric therapy of acute salmonellosis^43^. Furthermore, resistance of these strains to other therapeutic options including chloramphenicol and fluoroquinolones, leaves limited options for clinical management of severe infections. Our data were consistent with a distinct origin of an ESBL gene in Zimbabwe, unrelated to recent emergence of other ESBL genes in *S*. Kentucky DST198 in Europe and China. Similar *bla*_*CTX-M*_ genes to that identified in the Zimbabwe isolates reported previously were present in phylogenetically distinct clades and in a different genomic context. Most ESBL-producing strains from outside of Zimbabwe were associated with the *bla*_*CTX-M-14b*_ gene that differ from *bla*_*CTX-M-14.1*_ gene of some Zimbabwe isolates by a single amino acid substitution (I17F). The European center for disease control and prevention (ECDC) recently launched an Urgent Inquiry (UI-464) on a ciprofloxacin-resistant ST198 strain carrying a *bla*_*CTX-M-14b*_ gene conferring cephalosporin resistance integrated adjacent to the hcp1 gene on the chromosome^18^. This MDR clone of *S*. Kentucky ST198 is already widespread and has been declared a high-risk global MDR clone^17^. The strain spread to several EU countries^18,27^ but to date has only been reported in human infections^18^. In contrast, in China and ST198 clone carrying a chromosomally integrated *bla*_*CTX-M-14b*_ gene was isolated from a poultry slaughterhouse^44^. A second chromosomally encoded gene *bla*_VEB-8_ was identified in a *S*. Kentucky ST198^27^ and *bla*_CTX-M-15_ and *bla*_CMY_ genes carried on plasmids have also been reported in *S*. Kentucky ST198 isolates from Europe^27,28^. Further plasmid-mediated antibiotic resistance is concerning as plasmids may be more easily acquired during bacterial evolution, but may also be easily lost^45^.

A limitation of this study was the relatively small sample size of 37 *S*. Kentucky isolates analyzed. However, it already demonstrated the role that animals and humans in Zimbabwe play in the circulation of this emerging antimicrobial-resistant enteric pathogen. As far as we are aware this is the first study originating from Africa reporting on the presence of the epidemic ciprofloxacin-resistant ST198 with a novel ESBL *bla*_*CTX-M-14.1*_ gene located on an IncHI2 plasmid. Zimbabwe strains of ST198 exhibited a considerable increase in the number of genes from a median of nine to 18 AMR genes and conferring additional resistance to phenicols, phosphonic and extended-spectrum β-lactam antibiotics compared to MDR *S*. Kentucky reported previously^17,18^. The resistance profile is comparable to that described previously as extensively-drug resistance (XDR) in *S*. Typhi^46^ and has potentially significant implications to the clinical management of severe infections. The spread of ESBL-producing *Salmonella* serotypes is of great concern in many countries and the CTX-M family is the most common globally disseminated gene in a broad spectrum of microbial species^47^. The data highlight the need of an increased surveillance incorporating genomic epidemiology of NTS in both human and animal populations through a One Health approach. The information generated by continuous monitoring can be fed into policies and intervention to prevent the spread of this highly resistant clone and prevent the emergence of new ones.

## Methods

### Bacterial isolates used in this study

A total of 245 NTS strains isolated during routine surveillance by the National Microbiology Reference Laboratory of Zimbabwe were investigated in this study. Strains were isolated from human clinical infections (*n* = 162) during the period 2016 to 2020, chicken farms (*n* = 82) isolated from the years 2018 to 2020, crocodile meat (*n* = 1) and a dining table at a school (*n* = 1). The human *Salmonella* isolates (*n* = 162) were from stool (157/162) and blood samples (5/162) from clinical cases received from the National *Salmonella* Surveillance sentinel sites. Chicken farm isolates (*n* = 82) originated from chicken (*n* = 30), boot swab (*n* = 1), environmental swabs (*n* = 34), rectal swabs from asymptomatic farm workers (*n* = 10), litter (*n* = 1), and chicken feed pellet (*n* = 6) samples. Ethics approval for the study was granted by the University of Pretoria, South Africa (779/2018) and the Medical Research Council of Zimbabwe (MRCZ/A/2369). Strains are available upon request subject to requirements of the Nagoya Protocol.

### *Salmonella* isolation, serotyping, and antimicrobial susceptibility testing

*Salmonella* isolation, serotyping based on the Kauffmann–White–Le Minor scheme according to ISO 6579-1:2017^48^. Briefly, the test strain was cultured on Mueller Hinton (MH) agar and 2–3 colonies were suspended in sterile 0.45% saline on a glass slide. Antiserum (Mast, UK) was added and agglutination monitored for two minutes on a rocking plate. A control without antiserum was used to test for autoagglutination. Serotype was determined based in antigenic formula^49^. Antimicrobial susceptibility testing results using Kirby-Bauer disc diffusion assays were used as described previously^50^. Briefly, 4–5 colonies were resuspended in sterile 0.45% saline, turbidity adjusted to a 0.5 McFarland standard and inoculated onto MH agar with a swab. Antimicrobial discs impregnated with antimicrobial (Mast, UK) were placed on the surface and incubated at 35 °C for 18 h. The panel of antimicrobials tested comprised: ciprofloxacin (5 μg), ceftriaxone (30 μg), chloramphenicol (30 μg), tetracycline (30 μg), azithromycin (15 µg), ertapenem (10 µg), ampicillin (10 μg), ceftazidime (30 μg), ceftazidime + clavulanic acid (30 μg/10 μg), cefotaxime (30 μg), and cefotaxime + clavulanic acid (30 μg/10 μg) (Oxoid, UK). *Escherichia coli* ATCC 25922 was used as internal quality control. Results were interpreted using the Clinical and Laboratory Standards Institute (CLSI M100, 30th Edition) antimicrobial susceptibility testing standard (2020) included in WHONET 5.6 version software^51^.

### Whole-genome sequencing (WGS) and quality control

A volume of 1 mL of an overnight *Salmonella* culture in Tryptone Soy Broth (Oxiod, Hampshire, UK) was harvested by centrifugation for 2 min at 13,000 × *g* (ThermoScientific, Germany). Genomic DNA was extracted from the 245 *Salmonella* isolates using a Maxwell® RSC 48 automated nucleic acid purification instrument (Madison, Wisconsin, USA). The DNA concentration was measured with a Qubit fluorometer (Life Technologies, Carlsbad, CA, USA) and adjusted to 0.2 ng/µL, and stored at –20 °C before library preparation. The library preparation for short-read sequencing, was performed using the Nextera Flex DNA Library Preparation Kit according to the manufacturer’s instructions (Illumina, San Diego, CA, USA). Subsequently, sequencing was performed with a NextSeq benchtop sequencer (Illumina, San Diego, CA, USA). Raw sequence data were submitted to the Sequence Read Archive (SRA) (https://www.ncbi.nlm.nih.gov/sra) under study accession PRJNA762287. Read quality was assessed with fastp^52^ and summarized with multiqc^53^. Sequences with a theoretical read depth below 20x, or with less than 80% of reads attributed to *Salmonella* using Bracken were excluded from further analysis. Only samples that passed the quality control were considered for genomic analysis.

Freshly extracted DNA, for long-read sequencing, was ligated using native barcoding SQK-LSK109 following ONT recommendations. The library pool was loaded on a MinION Flow Cell (R9.4.1) at 43 fmol. The raw reads are available in the Sequence Read Archive (SRA) (accession PRJNA762287).

### Illumina short-read sequence analysis and assembly

The serotype formula 245 *S. enterica* strains isolated in Zimbabwe that passed the quality control were identified from short-read sequence data using SeqSero2^54^. Multilocus sequence type (MLST) for *Salmonella enterica*, the presence of antimicrobial resistance genes and plasmid replicon incompatibility group were identified in raw sequence reads using ARIBA^55^ with the ResFinder database^56^ or the plasmidfinder database^57^, with default settings. Raw sequence reads were assembled using SPAdes version 3.13.0^58^ and chromosomal point mutations in *gyrA, gyrB*, *parC*, and *parE* genes identified using RGI^59^. For phylogenetic analysis *S. enterica* strains isolated in Zimbabwe or 364 *S*. Kentucky ST198 genomes previously described^17,18,24,44^, raw sequence data were mapped to the reference genome strain 201001922 (GenBank accession number CP028357) using snippy version 4.1.0 (https://github.com/tseemann/snippy) with parameters (--mapqual 60 –basequal 13 –mincov 4 –minfrac 0.75) to identify single-nucleotide polymorphism (SNPs). Putative recombinogenic regions were detected based on SNP density and masked using Gubbins version 2.2.0^60^ with default settings. A maximum likelihood (ML) phylogenetic tree was built from an alignment of chromosomal SNPs, with RAxML^61^ version 8.2.8 using the GTR model with bootstraps as determined by the auto-mre flag. The tree visualized with ggtree^62^. HierBaps^63^ was used to estimate the population structure with a max depth of 3 and n.pops of 10. To investigate conservation of SGI1K using short-read data, reads were mapped to the SGI1K reference (genbank accession AY463797.8) using minimap2^64^ and the percentage of sequence covered assessed using bedtools^65^. The presence of individual SGI1K genes was assessed using ARIBA^55^.

### Long-read assembly and sequence analysis

Long-read data was assembled using trycycler^66^. The reads were filtered using filtlong (keep 95%, minimum length 1 kb) 12 subsamples of reads were generated, and assemblies generated using either flye, raven, or miniasm (four assemblies per software). Trycycler reconcile, and consensus was used to generate a consensus assembly. Pilon was used correct sequencing errors with matched Illumina short-read data, the quality of the polished assembly was assessed with QUAST^67^ and Socru^68^ was used to confirm the orientation of the chromosome fragments. The start of the chromosome was set to *thrL* using circlator^69^. BLAST was also used to compare SGI1-K to the genome assembly, and comparison of chromosomal and plasmids region of interest were visualized using genoplotR^70^. Plasmid taxonomic unit was identified using COPLA^71^ and compared against the plasmid database (PLSDB)^72^ using BLAST to identify closely related plasmids.

### Reporting summary

Further information on research design is available in the Nature Research Reporting Summary linked to this article.

## Supplementary information

REPORTING SUMMARY

Supplementary Information

Supplementary Data

## Data Availability

Sequence data reported for the first time in this study have been deposited in Sequence Read Archive (SRA) (accession PRJNA762287). All other sequence data used in the analysis are in available databases accessible using accession number (Supplementary Data).

## Code availability

All software used in the sequence analysis is freely available from repositories described in the cited literature.
