# Supplementary material for: Highly drug resistant clone of Salmonella Kentucky ST198 in clinical infections and poultry in Zimbabwe
Source: NPJ Antimicrob Resist. 2023 Jun 16;1:6. doi: 10.1038/s44259-023-00003-6 (PMC11721084; doi:10.1038/s44259-023-00003-6)
Supplement: Supplementary file 2 — Supplementary Information [file 44259_2023_3_MOESM2_ESM.docx]

**Supplementary Information**

**Highly drug resistant clone of *Salmonella enterica* serotype Kentucky ST198 in human clinical infections and poultry in Zimbabwe**

Tapfumanei Mashe^1,2,9‡^, Gaetan Thilliez^3,‡^, Blessmore V Chaibva^4^, Pimlapas Leekitcharoenphon^5^, Matt Bawn^3,14,15^, Moses Nyanzunda^6^, Valerie Robertson^7^, Andrew Tarupiwa^2^, Haider Al-Khanaq^3^, Dave Baker^3^, Moishe Gosa^6^, Marleen M Kock^1,8^, Stanley Midzi^9^, Mwamakamba Lusubilo Witson^10^, Matheu Jorge^11^, Jacob Dyring Jensen^5^, Frank M Aarestrup^5^, François-Xavier Weill^13^, Rene S. Hendriksen^5^, Marthie M Ehlers^1,8,#^ and Robert A. Kingsley^3,12,#^

1. University of Pretoria, Pretoria, South Africa

2. National Microbiology Reference Laboratory, Harare, Zimbabwe

3. Quadram Institute Bioscience, Norwich, United Kingdom

4. Ministry of Health and Child Care, Harare, Zimbabwe

5. Technical University of Denmark, Kgs. Lyngby, Denmark

6. Irvines, Harare, Zimbabwe

7. University of Zimbabwe, Harare, Zimbabwe

8. National Health Laboratory Service, Pretoria, South Africa

9. World Health Organization, Harare, Zimbabwe

10. World Health Organization Regional Office for Africa, Brazzaville, Republic of Congo

11. World Health Organization, Geneva, Switzerland

12. University of East Anglia, Norwich, United Kingdom

13. Institut Pasteur, Paris, France.

14. Earlham Insitute, Norwich, United Kingdom

15. Current address: University of Leeds, Leeds, United Kingdom

^‡^ These first authors contributed equally ^#^ These senior authors contributed equally

**Correspondence**: Tapfumanei Mashe [mashet2006@yahoo.co.uk](mailto:mashet2006@yahoo.co.uk) or Robert A. Kingsley [Rob.Kingsley@quadram.ac.uk](mailto:Rob.Kingsley@quadram.ac.uk)

Supplementary Figure 1. *In silico* prediction of serotype of *S. enterica* strains isolated from poultry farms and human clinical infections in Zimbabwe.


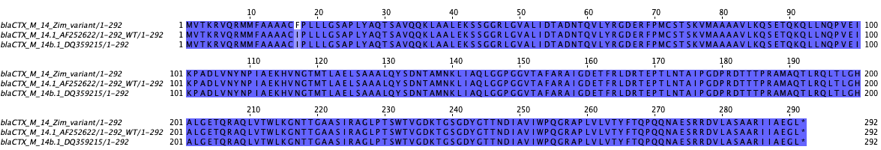


Supplementary Figure 2. Alignment of predicted amino acid sequences of extended spectrum beta lactamase encoded by *bla*_CTXM_ in plasmid pGTZIM1, *bla*_CTXM14.1_ and *bla*_CTXM14b.1_.

Supplementary Figure 3. Comparison of pGTZIM2 with a plasmid reported in strain NZ_CP053654.1. Open reading frames (blue boxes) and nucleotide sequence with >90% identity over >900bp (red shading) are indicated.

Supplementary Figure 4. Population structure of S. Kentucky ST198 strains isolated from Zimbabwe in the context of 364 globally sourced S. Kentucky strains. Colored boxes indicate the country of isolation and country implicated through recent travel, if known (inset key), and the presence of AMR genes are indicated (inset key).

Supplementary Data. Sequence data accession numbers and metadata for whole genome sequence of *Salmonella* strains used in this study. Available for download.
